# The right to traditional, complementary, and alternative health care

**DOI:** 10.3402/gha.v7.24121

**Published:** 2014-04-25

**Authors:** Maria Stuttaford, Sahar Al Makhamreh, Fons Coomans, John Harrington, Chuma Himonga, Gillian Lewando Hundt

**Affiliations:** 1 Division of Health Sciences, Warwick Medical School, University of Warwick, Coventry United Kingdom; 2 Department of Social Work, Al Balqaa Applied University, Fuhies-Alali, Jordan; 3 Faculty of Law, Centre for Human Rights, Maastricht University, Maastricht, The Netherlands; 4 School of Law, Cardiff University, Cardiff, United Kingdom; 5 Department of Private Law, Faculty of Law, University of Cape Town, Cape Town, South Africa

**Keywords:** right to health, plural health care, traditional, complementary and alternative therapies, legal basis, regulation, protection from harm, appropriate health care

## Abstract

**Background:**

State parties to human rights conventions and declarations are often faced with the seemingly contradictory problem of having an obligation to protect people from harmful practices while also having an obligation to enable access to culturally appropriate effective healing. As people increasingly migrate across the globe, previous distinctions between ‘traditional’ and ‘complementary and alternative medicine’ practices are being transcended. There are connections across transnational healing pathways that link local, national, and global movements of people and knowledge.

**Objective:**

This paper contributes to the development of the concept and practice of the right to health in all its forms, exploring the right to traditional, complementary, and alternative health (R2TCAH) across different contexts.

**Design:**

The paper draws on four settings – England, South Africa, Kenya, and Jordan – and is based on key informant interviews and a literature review undertaken in 2010, and updated in 2013. The paper begins by reviewing the international legal context for the right to health. It then considers legal and professional regulations from the global north and south.

**Results:**

Additional research is needed to establish the legal basis, compare regulatory frameworks, and explore patient and provider perspectives of regulation. This leads to being able to make recommendations on how to balance protection from harm and the obligation to ensure culturally appropriate services. Such an exploration must also challenge Western theories of human rights. Key concepts, such as individual harm, consent, and respect of the autonomy of the individual already established and recognised in international health law, could be adopted in the development of a template for future comparative research.

**Conclusions:**

Exploration of the normative content of the right to health in all its forms will contribute to supporting traditional, complementary, and alternative health service users and providers in terms of access to information, non-discrimination, clarification of state obligations, and accountability.

This paper sets out the need to explore the development of the right to health in relation to establishing the basis for a human right to traditional, complementary, and alternative health care (TCAH). People seek health in different ways, from biomedicine, traditional, complementary and alternative health care. Authoritative interpretation of the right to health contained in the International Covenant on Economic, Social and Cultural Rights (ICESCR) presents states with apparently contradictory obligations of protecting people from harmful practices on the one hand, and enabling access to culturally appropriate healing on the other.

For the purposes of this paper, we have adopted and extended the CAMbrella definition of complementary and alternative medicine (CAM). The CAMbrella project created a knowledge base and review of legal and lay understandings and use of CAM in Europe and as such provides a comprehensive starting point for future work. CAM is ‘an umbrella term for treatment practices mainly used outside conventional medicine. The most prominent CAM disciplines in the EU are herbal medicine, acupuncture, homeopathy and manual therapies (massage, chiropractic, osteopathy), but CAM also includes such practices as anthroposophic medicine and naturopathy. CAM is practised mostly in private practice by medical doctors and by practitioners trained in the specific disciplines’ (1). This definition, similar to many definitions of CAM, may be criticised for being a ‘negative definition’ in so far as defining CAM in relation to what ‘conventional’ or biomedicine is not (2). CAM has been considered to include therapies falling outside the sphere of biomedicine, but which are widely practiced outside of their country of origin (e.g. Chinese medicine, acupuncture, homeopathy, and massage). By contrast the term traditional medicine covers therapies that are predominantly practiced by people of the country where the therapy originated. Traditional medicine is often described as having religious and cultural significance (3, 4). These definitions fail to take into account the global mobility of people and knowledge about healing.

In the absence of global agreement on definitions of CAM and traditional healing, this paper extends the CAMbrella definition by also adopting the model of ‘integration’ in which there is an aspiration for collaboration, mutual respect, and expansion of existing dominant models to be genuinely holistic (5). Multidisciplinary and interprofessional collaboration exists with the aim of best serving the needs of the patient (5). Integration takes place at several levels ranging from the patient–healer level to health policy and health system level (6). CAMbrella's definition is also extended by being inclusive of traditional healing.

To date there has been a split in the definition of ‘traditional’ and ‘CAM’ that has been geographically based. Examples of south-south and north-south transfer of knowledge of healing practices are not new and as people increasingly move across the globe, ‘traditional’ is no longer only practised over ‘there’. Although the CAMbrella project focused on citizens’ access to CAM (7), the right to health is a fundamental right regardless of citizenship (8). Patients with both migratory and stable backgrounds will access TCAH (9). However, migrants’ decisions related to health and illness are situated and connect across transnational healing pathways that link local, national, and global movement of people and knowledge. Furthermore, the local lived experience of people shapes this knowledge (10–14). Although this often happens in the private sphere, decisions and actions do spill over into the public sphere, for example, sourcing food related to maintaining health across global boundaries (15, 16). This paper transcends previous distinctions between ‘traditional’ and ‘CAM’ practices to explore the right to health in all its forms across different contexts. By focusing on mobile populations, it is possible to be open to diverse modalities of healing without labelling them ‘traditional’ or ‘CAM’. While adopting an expanded definition of CAM to include traditional healing, we accept that for the purposes of law and regulation TCAH are currently defined negatively. State parties are obliged to simply ensure no harm from TCAH. In the future, research and recommendations for health policy and regulation could be cognizant of this.

This paper begins by identifying the gap in the current right to health policy and argues for the need for future research to establish the legal basis for the right to traditional, complementary, and alternative health care (R2TCAH), clarifying the existing right to health in all its forms. The paper further argues that future research needs to explore the legal and professional regulations from the global north and south to highlight opportunities for the development of regulatory frameworks. This will assist in addressing the contradiction between protecting from harm and promoting culturally appropriate care. A conceptual framework is needed to accommodate plural legal systems and plural health-seeking behaviour and the implementation of the right to health. In conclusion, it is proposed that a template for future comparative research could be developed to establish the normative content of the applicable law.

## Methods

The paper draws on four settings – England, South Africa, Kenya, and Jordan – and is based on a literature review undertaken in 2010, and updated in 2013, of policies and refereed papers published in English related to TCAH in these settings. In addition to the authors’ own research experiences of the right to health and of TCAH in these four contexts and from different disciplinary backgrounds, three key informant interviews were undertaken in South Africa and five in Jordan in 2010. The literature review and key informant interviews formed the basis of a seminar in 2010 in England between the co-authors, and in 2013, in Cape Town with a wider audience. This is supplemented by a review of legal materials and secondary literature as well as interviews with key informants conducted in Kenya in 2013.

The seminar in 2010 was used as a means to identify a focus on the topic of R2TCAH. The initial literature review sought to identify key themes in the area of traditional medicine; CAM; and the right to health. The aim of the key informant interviews was to gain an impression of the extent of importance of these issues for people who may be affected by future research or who might be able to use the future research. Prior to the 2010 seminar, the initial literature review and interview transcripts were circulated and reviewed by co-authors. Co-authors presented individual papers at the 2010 seminar and a focus on regulation and professionalisation was identified. The subsequent literature review focused on these areas.

The four countries make for relevant comparison because they are seeing acceptance of TCAH to varying degrees and are increasing regulation or attempts to regulate it. In England and South Africa, there are legal regulations governing biomedicine and certain CAM, with other CAM either professionally regulated or unregulated. In addition, in South Africa, there are legal regulations for traditional healing. In both contexts, there is evidence that people practice plural health seeking using a wide range of TCAH alongside biomedicine (14, 17). In Kenya, by contrast, only biomedicine is formally regulated and it remains dominant within the official health system. The last decade has seen a number of reform initiatives in relation to both CAM and traditional medicine, though none of these have yet resulted in legislation. Both Chinese medicine and alternative Western practices are increasingly popular among private patients in Kenya (18). Similarly, in Jordan, in private clinics people tend to use Chinese medicine, acupuncture, and reflexology. Indigenous people in Jordan visit secular and religious healers who use herbalism, read the Quran, and provide amulets and blessings. There has not been an explicit or official recognition of traditional healing or CAM. Al Makhamreh found that physicians and other health and social care professionals such as social workers would like more regulation of traditional practices and inclusion of information about CAM in health professional training (19).

## Results and discussion

The review of literature identified a gap in international human rights law and soft law (e.g. professional codes, guidelines, and patient charters) in terms of defining the right to health in all its forms beyond biomedicine. It was found that the lack of clarity on the legal basis for R2TCAH has resulted in weak legal and professional guidance and regulation in this area. Guiding principles for taking the research forward are existing protections and regulations, the right to information, the inclusion of equity and non-discrimination, and the notion of the universality of human rights. In addition our deliberations highlighted the need for research exploring ‘other’ healing modalities to also embrace ‘other’ forms of knowledge.

### The gap in international human rights law and soft law

This section sets out the gap in current international and regional human rights law and soft law and the need for future research to establish the legal basis for the R2TCAH, thereby further clarifying the existing right to health in all its forms.

The substantive issues relating to the implementation of the right to health were established by UN General Comment 14 on the right to the highest attainable standard of health (8) which explained and interpreted Article 12 of the UN ICESCRs (20). Article 12, a legally binding obligation for states that have ratified it, provides for the right of everyone to the enjoyment of the highest attainable standard of physical and mental health. Although not legally binding, General Comment 14 is a vital, authoritative, interpretative document for a more practical understanding of the right to health and its implementation. It is of significance, not only at the international level but also in a growing number of jurisdictions, such as South Africa and Kenya, which have adopted a justiciable right to health in their constitutions (21). The General Comment defines ‘the right to health *in all its forms*’ (8) (emphasis added) and includes several interrelated elements. These are an *available* functioning public and health care system in sufficient quantity, a physically and economically *accessible* health system free of discrimination with information readily accessible, an *acceptable* health system based on medical ethics, and a *culturally appropriate* and *quality* health system that is scientifically appropriate (8). The right to health places obligations on states parties to respect the right by refraining from direct interference or indirect interference by third parties of the enjoyment of the right. Furthermore, it places obligations on states to fulfil the right by adopting legislation, administration, budgets, promotional, and other procedures to realise the right to health (8). What is of particular interest to this research is that there is specific mention of *indigenous people* who ‘have the right to specific measures to improve their access to health services and care. These health services should be *culturally appropriate, taking into account traditional preventive care*, *health practices*, *and medicines*. States therefore should provide resources for indigenous people to design, deliver, and control such services … in indigenous communities, the health of the individual is often linked to the health of the society as a whole and has a collective dimension’ (8).

However, at the same time, it is considered a violation of the obligation to protect if states fail ‘to take all necessary measures to safeguard persons within their jurisdiction from infringements of the right to health by third parties. This category includes such omissions as the failure to regulate the activities of individuals, groups, or corporations so as to prevent them from violating the right to health of others; the failure to protect consumers and workers from practices detrimental to health, e.g. … the failure to *discourage the continued observance of harmful traditional medical or cultural practices*’ (8).

There is a significant tension between these two elements of General Comment 14, which can only be resolved through further interpretation of the right to health, which itself draws on studies of the manner in which traditional healing is practised.

Although there is growing research related to the quality of TCAH (22), the integration of TCAH into universal health systems remains controversial (5). In ratified and legally binding documents, the focus is more on the inclusion of TCAH in acute and emergency care (e.g. the International Convention on the Protection of the Rights of All Migrant Workers and Members of Their Families, 1990: Article 28), or on intellectual property rights and protection of the environment (e.g. Declaration on the Rights of Indigenous Peoples, 2007: Article 24 and Convention on Biological Diversity 1993). There are, however, a growing number of legal documents led by UNESCO (e.g. Declaration on the Human Genome and Human Rights, 1997; Declaration on Human Genetic Data, 2003; and Universal Declaration on Bioethics and Human Rights, 2005) that have an authoritative normative nature and include concepts agreed upon by states that will be of use when exploring the legal basis and developing a template for future research. These include individual harm, consent, and respect for the autonomy of the individual. For example, the 2005 Universal Declaration on Bioethics and Human Rights provides that ‘in applying and advancing scientific knowledge, medical practice and associated technologies, direct and indirect benefits to patients, research participants and other affected individuals should be maximized and any possible harm to such individuals should be minimized’ (article 4). In addition, any preventive, diagnostic, and therapeutic intervention must only be carried out with the prior, free, and informed consent of the person concerned (article 6). Finally, the declaration also emphasises the importance of cultural diversity, pluralism, and respect for human dignity in matters of bioethics and human rights (article 12). Violations of these norms entail the accountability of states and non-state actors.

General Comment 14 extends the reach of law to issues of health care delivery and governance and juridifies the activities of public health workers and corporations. It proposes an intensified juridification with its indication of a right to traditional medicine and the legal control of traditional healers. What would be the impact of this on practice and authority? Establishing the R2TCAH would strengthen the universal claim of the right to health as a human right by incorporating the cultural diversity of patients and treatments and recognise the different underlying values about health, illness, and treatment.

In summary, research is required to interpret the legal basis for the right to health in all its forms (*lex ferenda*) in the sense of contributing to the progressive development of the law as it should be, instead of relying only on the law as it currently exists (*lex lata*). In order to establish the legal basis, an analysis of domestic, regional, and international legal sources establishing the substance (normative content) and the justiciability of this right and human rights obligations needs to be undertaken. A template for future legal analysis drawing on individual harm, consent, and respect for the autonomy of the individual and the universality of human rights needs to be established which can then be used to support the implementation of the right to health in the context of TCAH.

### Implementation of the right to health in all its forms: the gap in domestic legal and professional regulations

This section identifies a gap in the legal regulations and argues that their future development will assist patients and providers in addressing the contradiction between protecting from harm and promoting culturally appropriate care.

#### Protecting from harm and the impact of regulation

The position of TCAH is changing. Globally, there has been a trend towards the integration of TCAH (23). There is growing recognition of plural health-seeking behaviour, cooperation between biomedical approaches, and TCAH and integrated care. This is being matched with increasing regulation. State obligations in relation to the R2TCAH are usually addressed in relation to protecting, fulfilling, and respecting access to biomedical forms of therapy. There is a growing body of research assessing the quality of CAM therapies, ethical issues related to beneficence and non-maleficence, defining intellectual property rights, and protecting the environment. However, there has been less research on the availability, accessibility, and acceptability of such therapies and the impact of increasing regulation on patient–provider relationships.

TCAH is subject to both voluntary and statutory regulation (24). Some countries have seen a liberalisation of TCAH regulations leading to a widening of access, but also growing concerns from patients as to what is ‘safe’. By contrast, others have seen increasing regulation (25). The latter may lead to greater availability of patient information about quality; however, there may also be increasing occupational closure and decrease in access (5). Where state and non-state actors engage with each other on the right to health, it is possible to start a ‘norm cascade’ in which dialogue and action can shift policies and rules (26). In this way, existing human rights practice can evolve in response to changing contexts. The normative content of the right to health can be clarified by reviewing regulations and engaging with patient and provider key informants to develop a template for future research.

#### The right to information

Common to all forms of healing – whether bio-medical or TCAH – is that patient needs are safely and ethically met 27. In particular, research has focused on the right of patients to information about all forms of care, patient choice, and freedom of thought and religion (28–32). Many of these ethical discussions lead to the central question of whether therapeutic results are beneficial or harmful, which is related to concepts of healing and health (2). Beyond the right to information, there is little discussion in the literature with regard to the R2TCAH, except for the recent call for regulation of non-conventional medicine included in a Framework Convention on Global Health (33). Although the right to information is identified in the literature and there is a growing acceptance of integrated care, research also shows that both patients and biomedical practitioners are cautious about discussing TCAH with each other (34–36). Research exploring the use of TCAH by migrants has identified particular challenges in relation to communication between patients and providers, which are exacerbated by language barriers and cultural differences (37–39). In the UK, medical students are required to learn about different healing systems and respect patient's wishes to consult different modalities of care. National Institute for Clinical Excellence guidelines emphasise the importance of patients telling their health care providers about the use of CAM. However, there may be reasons for patients being reluctant to do this (37). In a study on the use of biomedicine, CAM, and ethnomedicine for the treatment of epilepsy among people of South Asian origin in the UK, most participants had sought assistance from traditional South Asian practitioners and none of the people interviewed discussed this decision to use traditional therapies with their family doctor (4).

The right to information is integral to the right to health and is given particular emphasis in General Comment 14 (8). Future research will contribute to understanding how a rights based approach, which includes improving participation and accountability (40), can further support the patient–provider relationship through better access to information, improved communication (including disclosure by patients of their use of TCAH), and clarification about state obligations in relation to the right to information.

#### Equity and non-discrimination

There are issues of equity related to the practice of TCAH and the right to choose. Literature grounded in ethical arguments debates patients’ right to choose and the state's ability and taxpayers willingness to pay for treatments (7, 32). There is linkage between private and public spheres of decision making which are frequently gendered. In private spheres of decision making, such as households, where there are limited financial resources, women are more likely to access the cheapest form of treatment. For example, in India, where biomedical cancer care is expensive, women tended to use TCAH (41). In a recent study in Jordan of informal (family) care for people with mental health problems, it was found that issues of gender, reputation, stigma, and the cost of medicine led people to first access traditional medicine, then a biomedical general practitioner, and finally a mental health care service (19). People described how using traditional healing was easier to access and not stigmatised because of being embedded in indigenous cultural practice. Globally, where there is limited access to biomedicine, TCAH is the only form of healing and is essential to global health (23). In Kenya, those who consult traditional healers are usually poorer (42). However, other studies have found that users of TCAH usually have higher economic status (43). Those who consult CAM will probably be better off, given that this will be paid for out of pocket. Where biomedical approaches are provided by the state and TCAH is covered by out-of-pocket payments, the first choice of patients and households is often biomedicine (17). The right to health includes non-discrimination and equity as fundamental principles and future research will contribute to understanding how a rights-based approach can improve equitable access to TCAH and clarify the right to choose.

In summary, patient–provider relationships may be improved by exploring how the state obligation to discourage harmful practice is balanced with the state obligation to encourage culturally appropriate care. In terms of implementation, existing practises to ensure the right to information and non-discrimination need to be expanded to include consideration of the right to health in all its forms. An analysis of professional and legal regulatory frameworks in terms of the breach of state obligations to protect against individual harm and to ensure genuine patient consent will assist in this.

### Situating law, regulation, and health-seeking behaviour from the perspective of the universality of human rights

Much of the literature in the field under discussion is informed by Western theories of human rights [Sen (44) and Waldron (45)]. In order to explore the legal basis for the right to health in all its forms, especially for mobile populations, it is necessary to embrace plural legal frameworks and plural health-seeking behaviour. This section now considers African theories of rights and confronts the hegemony of Western theories of human rights by recognising that ‘another knowledge is possible’ (46).

The development of a human rights-based approach was rooted in the reality of particular contexts. The Vienna Declaration and Programme of Action of the Second World Conference on Human Rights (1993) confirmed that although human rights are universal, the significance of national and regional particularities and various historical, cultural, and religious backgrounds must be borne in mind. It has been argued that the most important aspects of a new human rights framework in Africa is that it be entirely democratic and launched in the interests of the people, and is straight-forward and unapologetic about its historical and social context (47). In this new ideology, African people are not helpless victims of rights abuses that need to be saved, but are the victors of their own struggle (47). In South Africa, through the recognition of a right to culture, there is a constitutional obligation for the courts to develop Indigenous Law and Customary law, on a case by case basis, so that they are aligned with the Constitution (48). Indigenous (customary) laws are not static by nature and South African constitutional jurisprudence has categorically confirmed this non-static, evolving, nature of customary law (49). These arguments have relevance to discussions around African traditional medicine as contemporary laws in Africa do not need to follow the same approaches as Western legal systems. The unique nature of Indigenous and African Customary Law in South Africa can make important contributions without conflicting with Western conceptions of human rights (48). The Kenyan constitution too expressly recognises culture as the foundation of the nation and commits the state to recognise the role of indigenous technologies in national development (Article 11 (1) and (2)(b)).

African philosophy exists as a continual discussion among Africans, for and within an African audience (50). To respond to contemporary challenges faced by African societies there should be the incorporation of the best of ‘Western heritage’ and the best of African ‘traditional knowledge’ (51). This recognises ‘constellations of knowledge’ (46) concerning the implementation of the right to health, plural healing, and plural legal systems but does not seek to valorise certain knowledge(s) (52). Gyekye (53) affirms that African philosophers have a role in providing conceptual responses to the challenges facing contemporary African societies within their current contexts. Furthermore, a conceptualisation of human rights in Africa should proffer culturally sensitive and legitimate rights without compromising the universality of rights (54). Reflections on the fundamental values and ethics that are grounded in the culture of the African people can facilitate this (53).

For novel work on human rights in Africa, grassroots experiences to formulate human rights frameworks and notions that resonate with African morals and belief systems can be developed and be accepted and useful in practice. Gyekye's insights suggest that we do not need to reject all lessons from philosophies of non-African origin. However, African human rights theorists must critically examine Western notions of human rights to see if and how they apply to African socio-political life. This implies that there may be some universal components of human rights, but that these should be used to deepen our understanding of African perspectives on human rights. As An-Na'im and Hammond have put it ‘the way to get a universal idea [right to health as a human right] accepted locally is to present it in local terms, which can best be done by local people. Conversely, local acceptance enriches the universal idea … by giving it meaning and relevance to people's lives’ (55).

## Conclusions

This paper has argued for the need to explore the global interdependence and transfer of knowledge of TCAH as a contribution to establishing the legal basis for recognition of the R2TCAH as a human right. There is currently a gap in the right to health in the context of plural health-seeking behaviour. Future research drawing on concepts of individual harm, consent, and respect for the autonomy of the individual and the universality of human rights will contribute to optimising the delivery of health care to people by exploring the appropriate use of health therapies and how health care practitioners and users can balance this with quality care. Such research will contribute to improving the patient-provider relationship. At a policy level a review of Patient's Rights Charters, or similar charters, from the perspective of the right to health in all its forms would identify how both patients and providers can use existing professional and patient guidelines to improve information about and experiences of the R2TCAH. Future research may clarify the normative content of law, legal, and professional regulations, and transcend previous distinctions between ‘traditional’ and ‘CAM’ practices. This future research will contribute to understanding how a rights-based approach can better support patients and providers in terms of access to information, non-discrimination, clarification of state obligations, and accountability. Such an exploration must also challenge Western theories of human rights. Clarifying the normative content of the right to health in all its forms, contributes to how states, policymakers, and providers may support TCAH service users as they are faced with a seemingly contradictory problem of guarding against harmful practices while encouraging culturally appropriate healing.
